# In Situ Investigation of Interrelationships Between Morphology and Biomechanics of Endothelial and Glial Cells and their Nuclei

**DOI:** 10.1002/advs.201801638

**Published:** 2018-11-10

**Authors:** Gonzalo Rosso, Ivan Liashkovich, Victor Shahin

**Affiliations:** ^1^ Biotechnology Center Technische Universität Dresden Tatzberg 47/49 01307 Dresden Germany; ^2^ Institute of Physiology II University of Münster Robert‐Koch Str. 27b 48149 Münster Germany

**Keywords:** atomic force microscopy, cell mechanics, endothelial cells, nanotechnology, Schwann cells

## Abstract

Morphology and biomechanics of cells and nuclei are interlinked with one another and play key roles in fundamental physiological processes. While powerful approaches are available for performing separate morphological and biomechanical investigations on cells and nuclei, simultaneous investigations in situ are challenging. Here, an appropriate approach is presented based on the simultaneous combination of atomic force microscopy and confocal microscopy in situ. Two cell types with entirely different morphologies, physiological roles, and biomechanical environments are investigated: vascular endothelial cells (ECs) with dense cytoskeletal actin, and nervous system glial cells (Schwann cells (SCs)) with dense vimentin network. Results reveal that ECs and their nuclei show high pliability and tend to undergo deformation only at compression sites. SCs, in contrast, show greater ability to resist mechanical deformation. Likewise, SC nuclei are harder to deform than EC nuclei, despite that SC nuclei have significantly lower amounts of lamins A/C, which reportedly scale with nuclear stiffness. The morphology–biomechanics interrelationships in SCs, ECs, and their nuclei may be a key factor in ensuring their physiological functions. In adult SCs, mechanosensitivity is presumably traded for mechanical strength to protect the neurons they encase, whereas ECs maintain mechanosensitivity to ensure specific local physiological response to mechanical stimuli.

## 1 Introduction

Cells are generally embedded in tissues and are exposed to a variety of physical forces arising from their immediate physiological surroundings.^1^ The nature of the forces varies among different cell types and species. For instance, chondrocytes and myocytes are exposed to compressive and tensile forces, respectively.^2^, ^3^ Neurons and the glial cells of the peripheral nervous system, Schwann cells, are exposed to both compressive and tensile forces.^4^, ^5^, ^6^, ^7^ Meanwhile, endothelial cells and vascular smooth muscles cells surrounding the endothelium are subject to shear stress and circumferential stretch.^8^

The ability of cells to sense their mechanical surrounding is known as mechanosensing, a physiological process that is utilized by cells and tissues in multiple stages during their lifecycle.^9^ Mechanosensing triggers specific and prompt biochemical signaling that is transmitted to the nucleus within microseconds, eventually leading to the regulation of mechanoresponsive genes.^10^ This intimate crosstalk between mechanical and biochemical cues is a key factor in the regulation of embryogenesis, development, differentiation, proliferation, adhesion, and migration, amongst others processes.^11^, ^12^, ^13^, ^14^

It is well understood that numerous cellular components are engaged in mechanosensing and transmission of the mechanical signal.^9^, ^15^, ^16^ For instance, local forces may be perceived by mechanosensitive channels, integrins, or focal adhesion proteins, amongst other players.^16^ The signal is then passed on to the cytoskeleton and associated components such as motor proteins and α‐actinin.^10^ Subsequent transmission of the signal from the cytoskeleton to the nucleus requires linker of nucleoskeleton and cytoskeleton (LINC), which includes nesprins, sun and lamin proteins.^10^ The LINC complex can be thought of as a bridge that physically connects the cytoskeleton with the nucleoskeleton, thereby facilitating direct nucleo‐cytoskeletal coupling. It relays and focuses the propagating mechanical signal onto underlying specific gene sites, eventually giving rise to regulation of gene expression.^10^ As the nucleus is the final destination for these signals and not directly exposed to the exterior of the cell, it was initially overlooked as a mechanosensor. However, the indications are that the largest intracellular organelle may indeed act as a specific cellular mechanosensor.^12^, ^17^, ^18^

Considered from simplified biophysical aspects, the nucleus accommodates a number of components that provide structural and mechanical support not only to the nucleus itself but also, because of its size, to the cell as a whole. The nucleus is separated from the cytosol by two phospholipid‐bilayer membranes, an outer and inner nuclear membrane facing the cytosol and the nucleus, respectively.^19^ The two largely separated membranes are joined at regular distances by nuclear pore complexes that mediate all nucleocytoplasmic transport.^19^ Together, the entity of the nuclear membranes and nuclear pores is known as the nuclear envelope, which in many species, including humans, contains another component designated the nuclear lamina.^19^ The nuclear lamina is a filamentous network of proteins which lines the inner nuclear membrane.^20^ It is referred to as the nucleoskeleton and is considered to be the primary determinant of nuclear mechanics and stiffness.^21^, ^22^ Its major constituents are type V intermediate filaments consisting of different types of lamin proteins.^21^ Accessory structural proteins in the nucleoskeleton, including titin and αII‐spectrin, lend additional support to the nuclear lamina.^21^ The nuclear interior, containing chromatin, is believed to be a further component affecting nuclear mechanics in both healthy and diseased cells.^23^, ^24^

Nuclear mechanics is a subject of particular interest owing to its fundamental importance to the overall morphology, dynamics, and physiological functions of cells.^25^ Moreover, impaired nuclear mechanics is closely linked to numerous diseases with severe consequences to the afflicted individuals.^25^ However, while there seems to be a close relationship between shape and mechanics of the nucleus, this relationship is incompletely understood; as are its implications for overall cellular mechanics and function. Elegant methods are available for investigating single cell and nuclear mechanics from distinct aspects, including micropipette aspiration,^26^, ^27^ optical tweezers,^28^ nuclear strain,^29^ real‐time deformability cytometry,^30^ and quite frequently atomic force microscopy (AFM).^6^, ^31^, ^32^, ^33^ AFM combines several advantages that render it a powerful approach to study cellular and nuclear mechanics at high resolution in physiologically relevant environments, especially when combined with confocal fluorescence microscopy which can be used simultaneously to assess cell and nuclear morphology.^5^, ^23^, ^34^, ^35^, ^36^ Thus, in the present work we apply a combined AFM‐confocal setup to study in situ the interrelationships between the morphology and biomechanics of cells and nuclei using two distinct cell types that are naturally exposed to different physical forces and mechanical surroundings and fulfill vastly different physiological roles: Schwann cells (SCs) and vascular endothelial cells (ECs).

## 2 Results and Discussion

The primary SCs utilized in the present work are harvested from sciatic nerves. The ECs utilized, EA.hy926 cells, are widely used for vascular endothelial cell research. This permanent endothelial cell line is derived by fusing primary human umbilical vein endothelial cells with the permanent human cell line A549,^37^ such that this fused line preserves the properties of the primary cell line.

**Figure 1:**
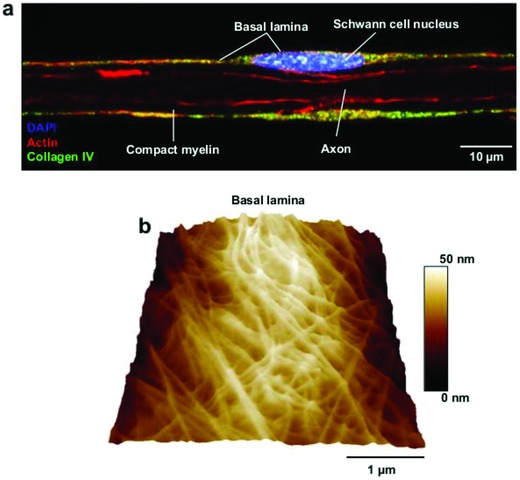
The SC nucleus is mechanically compressed in native myelinated nerve fibers. a) Representative confocal image showing a mouse adult isolated nerve fiber labeled against basal lamina collagen IV (green), the filamentous actin (red), and the nucleus (blue). b) The collagen‐rich basal lamina (topographical AFM image) and the axonal cytoskeleton impose some mechanical compression onto the SC nucleus (flattened shape).

SCs and ECs differ in their physiological roles as well as in their anatomical and mechanical surroundings. SCs produce myelin, which facilitates the physiologically critical high conduction velocity of nerves. In addition, they play key roles in the development, protection, maintenance, survival, and regeneration of the peripheral nervous system^38^; within peripheral nerves, they are tightly surrounded by diverse tissues. At the same time, they are exposed to compressive and tensile forces during the regular physical activities that engage muscles and nerves throughout daily life. SCs get some mechanical support from the basal lamina,^5^, ^39^ a special form of extracellular matrix that also plays key roles in mediating SC functions and their interaction with the microenvironment. As seen in **Figure** **1**, SCs reside in a confined space around the axons of peripheral nerves. The inward‐directed pressure against SCs as a consequence of the tight outer space confines the internal space of the cell and leaves little room for the nucleus. This physical stress apparently exposes the nucleus to some mechanical compression, judging from its rather flattened shape.

**Figure 2:**
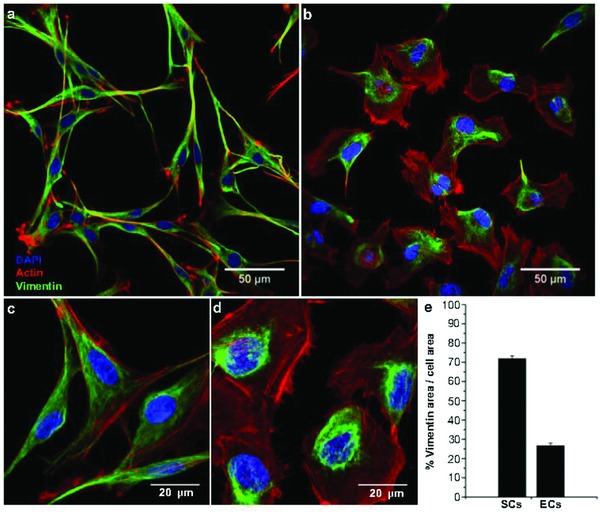
Spreading morphology and vimentin network distribution in isolated SCs and ECs. Representative confocal images showing a,c) typical elongated SC and b,d) multipolar EC spreading morphologies. Cell were stained with the dyes 4′,6‐diamidino‐2‐phenylindole (DAPI, blue) and Alexa Fluor 647 Phalloidin (red) for nucleus and F‐actin labels, respectively. Vimentin intermediate filament network was stained with anti‐vimentin antibody (green). c,d) Images at higher magnification. e) Percentage of vimentin network area/cell area ratio for SCs (*n* = 50) and ECs (*n* = 50).

In contrast to SCs, vascular ECs generally enjoy a larger space inside the blood vessels from their apical side and are exposed to other types of mechanical stresses. **Figure** **2** shows representative confocal microscopy images of isolated SCs and ECs (EA.hy926), wherein cell nuclei (blue), F‐actin (red), and intermediate filament vimentin network (green) are visualized following staining with 4′,6‐diamidino‐2‐phenylindole (DAPI) (Sigma‐Aldrich, USA), Phalloidin (Invitrogen, USA) and an anti‐vimentin (Sigma‐Aldrich, USA) antibody, respectively.

Both cell types reveal multiple morphological differences that are summarized as mean ± SEM values in **Table** **1**, based on measurements carried out on 50 cells each. The EC height and area are 15.88 ± 0.47 µm and 1805.90 ± 82.10 µm^2^, respectively, and are thus significantly greater than SC height (8.96 ± 0.89 µm) and area (1294.17 ± 90.80 µm^2^). The same is true for EC nuclei height (12.73 ± 0.59 µm) and area (234.91 ± 6.30 µm^2^) compared to SC nuclei height (7.45 ± 0.66 µm) and area (126.14 ± 5.50 µm^2^). Comparison of height‐to‐width ratios in EC and SC nuclei also yields markedly higher values for ECs (0.78 ± 0.12) compared to SCs (0.54 ± 0.15). In contrast, the nucleus height‐to‐cell height ratio in ECs is 0.80 ± 0.78, which is slightly lower than the counterpart ratio in SCs of 0.83 ± 0.70.

**Table 1:** Summary of cell and nuclei morphometrical data analysis

	Schwann cells	Endothelial cells
Cell height [µm]	8.96 ± 0.89	15.88 ± 0.47
Cell area [µm^2^]	1294.17 ± 90.80	1805.90 ± 82.10
Nucleus height [µm]	7.45 ± 0.66	12.73 ± 0.59
Nucleus area [µm^2^]	126.14 ± 5.50	234.91 ± 6.30
Nucleus width [µm]	13.72 ± 0.99	16.14 ± 0.88
Nucleus height/width	0.54 ± 0.15	0.78 ± 0.12
Nucleus height/cell height	0.83 ± 0.70	0.80 ± 0.78
*n*	50	50

Particularly striking is the difference in the amounts of the cytoskeletal components vimentin and F‐actin between SCs and ECs. Abundance of F‐actin is common in several cell types including ECs, whereas vimentin appears to dominate in SCs based on fluorescence staining in Figure 2d. This is supported by the analysis of the vimentin area‐to‐cell area ratio in SCs compared to ECs. The SC and EC vimentin areas are 71.9 ± 1.4 µm^2^ (*n* = 44) and 26.7 ± 1.35 µm^2^ (*n* = 44), respectively.

We hypothesized that the overall morphological differences between SCs and ECs are closely associated with differential mechanical properties, and thus we set out to corroborate this hypothesis. For this purpose, we designed an assay enabling measurement of cellular and nuclear mechanics at high resolution in situ based on a simultaneous combination of AFM and confocal microscopy. While in situ measurements of nuclear mechanics are challenging, they do offer a key advantage over measurements on isolated nuclei. Isolated nuclei are deprived of their complex natural biochemical and mechanical surroundings inside the cell and may not resemble the real behavior of nuclei in situ. When isolated, nuclei are loose and free of mechanical stresses exerted by cytoskeletal components, the LINC‐complex and several others physiological factors; we deemed this fact important in our consideration of nuclear mechanics.

**Figure 3:**
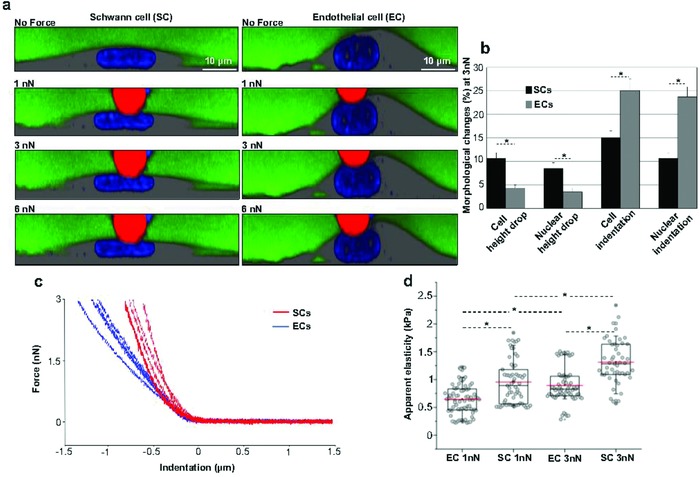
Simultaneous AFM‐confocal microscopy measurements to investigate in situ cellular‐nuclear biomechanics and structure interrelationships on living SCs and ECs. a) Visualization of the mechanical response of a SC and an EC before compression (No Force) and at increasing compression forces (1, 3, and 6 nN) with a 10 µm AFM bead (red). Cells are immersed in a medium containing 70 kDa‐dextran FITC (green) to visualize cell shape and control the functional integrity of cells and nuclei. Nuclei are stained with Hoechst (blue). b) Graph showing percentage drop in cells and nuclei heights (50 cells and nuclei each type) mechanically compressed at 3 nN loading force. c) Representative single force‐indentation curves obtained on SCs (*n* = 4, red lines) and ECs (*n* = 4, blue lines) at 3 nN loading force. d) Graph shows the apparent elasticity (Young's moduli) obtained for ECs and SCs at 1 nN (EC = 77 cells; SC = 61 cells) and 3 nN (EC = 65 cells; SC = 51 cells). The data are shown in a box‐and‐whiskers plot whereby the median value is represented by the red line and the borders of the boxes represent 25% and 75% percentiles, with a whisker range of 10%–90%. (*P < 0.05, b) *t*‐test, d) Mann–Whitney test (nonparametric groups).

In the utilized assay, nuclei are visible following staining with Hoechst (Sigma‐Aldrich, USA). Cells are kept in a medium containing 70 kDa fluorescein isothiocyanate (FITC)‐dextran, which serves two distinct functions: it renders the cells visible and acts as a functional integrity marker of cells and nuclei. FITC‐dextran remains fully excluded from cellular and nuclear entry unless cells/nuclei lose their structural, mechanical, or functional integrity, such as when they are exposed to mechanical stresses exceeding their limits. The advantage of simultaneous AFM‐confocal combination lies in the fact that the cell and nucleus structure and deformation can be visualized immediately in response to incremental loading forces exerted by the red‐fluorescent AFM bead. Moreover, as the nucleus is labeled, its response to mechanical forces can be studied separately from the overall cellular response. This is particularly important if one wants to determine whether or not the nucleus is deformed while the cell is exposed to incremental loading forces. **Figure** **3** summarizes several of this study's investigations of cellular‐nuclear mechanics, structure, and interrelationships.

Figure 3a shows how SCs, ECs, and their nuclei respond following exposure to loading forces of 1, 3, and 6 nN. Figure 3b summarizes the response based on measurements of 50 cells each at 3 nN. Cell height drop is significantly greater in SCs (10.72 ± 1.14%) compared to ECs (4.51 ± 0.73%). Likewise, nuclear heights drop by 8.53 ± 1.22% and 3.54 ± 0.81% in SCs and ECs, respectively. In contrast, cell indentation, which is considered as the locally induced penetration depth of the AFM bead, is significantly greater in ECs (25.15 ± 2.41%) compared to SCs (15.10 ± 1.42%), and the same is true for their nuclei (23.72 ± 2.10% and 10.73 ± 1.13%, respectively). These observations indicate that SCs and their nuclei tend to counter local deformation, which is the opposite of what is seen for ECs and their nuclei. In addition, we find differences in the force‐indentation curves shapes obtained after compressing both cell types at the same loading force (Figure 3c). Force‐indentation curves from ECs (blue lines) show a shallow slope in comparison to those from SCs (red lines). Further, the elasticity analysis shown in Figure 3d reveals that SCs possess a higher apparent Young's modulus (957.3 ± 51.2 Pa at 1 nN; 1311.2 ± 57.6 Pa at 3 nN) compared to ECs (642.8 ± 31.9 Pa at 1 nN; 894.2 ± 39.1 Pa at 3nN). When ECs were exposed to forces exceeding 6 nN, we frequently observed the formation of blebs, common indicators of apoptosis, suggesting a mechanical failure as a consequence of excessive stress. The applied range of forces was therefore limited to up to 6 nN.

It seems that when exposed to these forces, SCs and their nuclei swiftly spread the locally exerted stress and therefore evenly flatten, while ECs and their nuclei tend experience the deformation only at the site of exposure. This behavior implies that SCs have a mechanoprotective response whereas ECs have a mechanosensitive response. Indeed, even though SC progenitors during embryonic development rely strongly on their mechanosensitivity to interact with their mircroenvironment and promote nerve growth, mature SCs trade it for mechanoprotection of the vulnerable axons inside the mature nerve.^5^, ^39^

For ECs, their assumed mechanosensitive response is also in strong agreement with their well‐known functions. The vascular endothelium is a thin layer of cells that line the inner side of blood vessels and perform several physiological tasks. They release specific molecules that are utilized to contract or dilate blood vessels in accordance with the demands of blood flow. They also release other molecules which play key roles in platelet function and blood clotting as well as immune system regulation. Mechanosensitivity of ECs and nuclei is likely to be of key importance to facilitate a specific response to a locally induced mechanical load. In other words, we think that ECs sense and direct the local mechanical signal through a confined route. They transmit mechanical signals through cytoskeletal and other components to specific nuclear sites and eventually to underlying mechanosensitive genes; such behavior would prevent diffuse and unspecific signaling.^12^, ^40^ This consideration is supported by two simultaneous observations. The total heights of ECs and nuclei remain largely unchanged, while the indentation sites of cells and nuclei are confined to the site of exposure and are closely matched.

We also assume that these same observations provide direct experimental support for the emerging view that the nucleus is a cellular mechanosensor.^12^ The action of nuclei as mechanosensors may be limited to cell types that require specific responses to physiological mechanical stress, for instance ECs. Mature SCs nuclei, on the other hand, should primarily provide critical mechanical and structural strength, and mechanosensitivity would be counterproductive and therefore traded for more useful functions. Moreover, it seems plausible that a nucleus of a particular cell type could transform from a mechanosensor to a mechanoprotector in accordance with the cellular demands, for instance during developmental stages and differentiation. This would require a close tuning between structure and mechanics of cells and nuclei.

It is still unclear what determines the different types of mechanical–structural relationships and behaviors seen in SCs, ECs, and their nuclei. Cytoskeletal components are known to be major contributors to overall cellular mechanics and they also affect nuclear mechanics to some degree, but the more prominent contributor to nuclear mechanics is believed to be the nuclear lamina, in particular lamins A/C, which directly scale with nuclear stiffness.^12^, ^22^ Mutation and disruption of lamins A/C are closely associated with several diseases known as laminopathies.^12^

**Figure 4:**
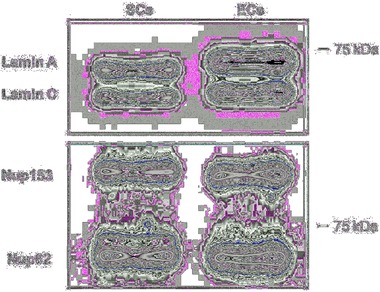
Nuclear intermediate filament protein lamin A/C expression in SCs and ECs. Western blot shows markedly increased levels of lamina A/C in ECs compared to SCs. The nuclear pore complex proteins Nup153 and Nup62 are used as controls.

Surprisingly, **Figure** **4** shows that expression levels of lamins A/C are significantly higher in ECs compared to SCs. While this observation may appear puzzling at first glance, we believe that it is not at all in disagreement with previous observations. The mechanical strength provided by the nuclear lamina is observed in nuclei of skeletal and cardiac muscle cells that are exposed to regular contraction. We therefore envisage that different isoforms of lamins A/C with different mechanical properties are expressed in different cell types depending on the functional demands. The cytoskeleton of SCs is strikingly rich in vimentin compared to the F‐actin‐rich ECs, and Figure 2 shows that vimentin is spread all over the cell, thereby shielding the SC nucleus. We therefore assume that vimentin contributes significantly to the mechanics of SCs and their nuclei. SCs vimentin plays key physiological roles in the peripheral nervous system, such as in regulating myelination,^41^ and vimentin networks greatly contribute to the overall cell mechanics and possess characteristic and unusual viscoelastic properties that are not shared by the other cytoskeleton components actin and tubulin.^42^ Thus, the differences in the content of actin and vimentin in SCs and ECs may affect not only their overall mechanics but also their structures. These differences may also determine whether a nucleus acts as a mechanosensor or mechanoprotector.

## 3 Conclusions

The cell nucleus has been described as a mechanosensor and is certainly important in the physiological function of many cell types. Based on the collected data, we conclude that SCs and ECs behave morphologically and mechanically different when exposed to physical stress. We propose that vimentin filaments provide mechanical protection to SCs and their nuclei against compression, whereas ECs and their nuclei are more compliant and nuclear deformation occurs at the site of compression, such that ECs show a localized mechanosensitive response. It is conceivable that for SCs, mechanical resistance is important in the peripheral nerve microenvironment to protect crucial axons and withstand mechanical loads from the adjacent stiff basal lamina. Conversely, in ECs, a mechanosensitive phenotype could help promote their sensitivity to the mechanical stimuli present in the vasculature. Nonetheless, the mechanism underlying the interplay between morphology and biomechanics of cells and their nuclei should be investigated further, as comprehensive investigations are necessary to determine in more detail which molecular complexes favor mechanoprotection over mechanosensing in glial and endothelial cells. This will contribute to a better understanding of their cellular and nuclear physiology, their mechanobiology, and associated diseases.

## 4 Experimental Section

*Isolation and Preparation of Myelinated Nerve Fibers*: All of the animal experiments were carried out in accordance with the European Convention for Animal Care and Ethical Use of Laboratory Animals following approval by the local governmental authorities (State Office for Nature, Environment and Consumer Protection, North Rhine‐Westphalia, Germany; File reference 84‐ 02.05.20.12.146). Peripheral nerve fibers were obtained from isolated sciatic nerves of adult C57BL/6 mice. For this purpose, animals were euthanized by isofluoran overdose and cervical dislocation. Forceps were used to harvest parts of both sciatic nerves (≈1 cm) which were immediately placed in ice‐cold PHEM buffer (25 × 10^−3^ m Hepes, 10 × 10^−3^ m EGTA, 60 × 10^−3^ m Pipes, 2 × 10^−3^ m MgCl2, pH 7.4). After removal of the epineurium, individual nerve fibers were mechanically separated on a poly‐l‐lysine‐coated glass surface and fixed immediately with ice‐cold 4% paraformaldehyde for 20 min. Prior to subsequent immunohistochemistry analysis, samples were washed three times and kept in PHEM buffer.

*Nerve Fiber Surface Topography*: AFM (Nanowizard 3 AFM system, JPK instruments, Germany) was applied to image individual sciatic nerve fibers in PHEM buffer. Imaging was performed in contact mode using a triangular V‐shaped MSCT silicon nitride cantilever (Bruker, USA) with an ≈0.6 N m^−1^ spring constant, 85 mm length, and 18 mm width. JPK data analysis software (JPK, Germany) was utilized for image processing.

*Primary Schwann Cell Purification and Culture*: Adult rats (Sprague–Dawley) were euthanized by an overdose of isofluoran followed by guillotine decapitation. Sciatic nerves were removed and placed in ice‐cold Dulbecco's modified Eagle's medium (Life technologies, Germany) containing 5% fetal bovine serum (FBS HyClone, GE Healthcare, USA), 100 µg mL^−1^ penicillin/streptomycin and 100 µg mL^−1^ glucose. Epineural tissue was removed to reduce fibroblast contamination. Nerves were cut in small pieces and transferred to a sterile culture petri dish containing culture medium and kept in incubator for several days. After 7 d the tissue was biochemically dissociated with collagenase and dispase (Sigma‐Aldrich, USA) followed by mechanical trituration. Dissociated nerve tissue was plated and incubated for 24 h at 37 °C and 5% CO_2_. On day 8, the nerve tissue was mixed and cells were centrifuged for 3 min at 250 × *g* and suspended in DMEM medium containing 10% FBS (HyClone), 2 × 10^−6^ m Forskolin (Sigma‐Aldrich, USA), 10 ng mL^−1^ Neuregulin (Peprotech, Germany), 10 µg mL^−1^ bovine pituitary extract (Sigma‐Aldrich, USA), and 100 µg mL^−1^ glucose. SCs were plated on poly‐l‐lysine‐coated culture flasks and incubated at 37 °C and 5% CO_2_ for 48 h. On day 10, SCs were harvested using trypsin and centrifuged for 3 min at 250 × *g* and kept in T‐25 culture flasks until reaching confluence. SCs were separated from fibroblasts applying the cold‐jet technique as previously described.^43^ Two more rounds of cold‐jet and plating were applied to increase SC purity. SCs in the experiments were between passages 4 and 7.

*Endothelial Cell Culture*: EA.hy926 endothelial cells were cultured at 37 °C, 5% CO_2_ minimal essential medium (MEM) supplemented with 1% nonessential amino‐acids, 1% MEM vitamins (Invitrogen), penicillin (100 µg mL^−1^), streptomycin (100 µg mL^−1^), and 10% fetal calf serum (FCS, PAA, Germany). ECs were grown until confluence on poly‐l‐Lysine coated T‐25 culture flasks. ECs in the experiments were between passages 10 and 20.

*Simultaneous In Situ AFM‐Confocal Measurements to Investigate Cell and Nuclear Mechanics*: SCs and ECs were plated separately on laminin‐coated 35 mm glass‐bottom petri dishes (WillCo, The Netherlands). All measurements of overall cell mechanics (including nuclear mechanics) were performed in situ on living cells, that is on cells containing their nuclei. For this purpose, we used an AFM (Nanowizard 3 AFM system, JPK, Germany) coupled to a laser scanning confocal microscope Leica TCS SP8 (Leica, Germany) equipped with an HC PL Apo 633 oil NA 1.40 lens and 488, 543, and 633 nm lasers lines. The simultaneous AFM‐confocal combination allows the investigation of overall SC and EC mechanics at the nanoscale level while providing real‐time images of cell and nucleus deformation at controlled forces. A 10 µm polystyrene red‐fluorescent bead glued to the end of a tipless cantilever (kc = 0.01–0.06 N m^−1^, Novascan, USA) was utilized to indent SCs and ECs. Fluorescent images of transversal sections of cells and their nuclei at different loading forces (1, 3, and 6 nN) were obtained using a bead‐cell extension delay of ≈20 s. Special care was taken to precisely place the bead over the center of the cells and their nuclei to avoid undesired compression of the edges. A blue‐fluorescent marker (Hoechst Sigma‐Aldrich, USA) was used to image and measure the nuclear height and indentation. In addition, 1 mg mL^−1^ 70 kDa FITC‐Dextran was mixed in the measurement buffer (HEPES: 140 × 10^−3^ m NaCl, 5 × 10^−3^ m KCl, 1 × 10^−3^ m MgCl_2_, 1 × 10^−3^ m CaCl_2_, 5 × 10^−3^ m glucose, and 1 × 10^−3^ m l‐arginine, pH 7.4) to visualize and measure the cell height and indentation before and after compression. Fluorescent images were acquired with the Leica Application Suite Advanced Fluorescence (LAS AF) software (Leica, Germany). Z‐stacks of ≈20 frames of 0.5 µm spacing at a resolution of 2024 × 32 pixels were generated for each cell. The transversal views of SCs and ECs were produced by mounting the *z*‐planes and then rotating them 90° using LAS Montage 3D viewer tool (Leica, Germany). For comparison between the apparent Young's modulus of SC and EC nuclei, the bead‐sample extension delay was set to 0 s. The contact point on each force curve and the apparent elastic modulus of SC and EC nuclei were determined with the Hertz model corrected for spherical indenters utilizing the JPK data processing software (JPK, Germany). For a spherical indenter, the bead‐sample force (*F*) is given by the following mathematical equation

(1) $$F\;=\;\frac{4E\cdot R^{\frac{1}{2}}}{3\left(1-v^{2}\right)}\cdot \delta^{\frac{3}{2}}$$

where *E* is the elastic Young's modulus, *v* is the Poisson's ratio of the sample (*v* = 0.5 value commonly used for cells), δ is indentation depth, and *R* is the radius of the sphere used to compress the nucleus (*R* = 5 µm for our measurements). Elasticity values were obtained fitting the full indentation range of each force curve. The sample temperature was controlled and kept at 37 °C throughout the experiment. During data acquisition the tip velocity was set to 1 µm s^−1^ to reduce the hysteresis.

*Immunohistochemistry*: Teased sciatic nerve fibers were incubated with blocking buffer (phosphate buffered saline (PBS) + 5% normal goat serum). A polyclonal antibody against collagen‐IV (1:200 Acris, Germany) was used to visualize the SCs basal lamina. The actin cytoskeleton and nuclei were labeled with Alexa Fluor 647 Phalloidin (1:100, Invitrogen) and DAPI (Sigma‐Aldrich, USA), respectively.

SCs and ECs were cultured over laminin‐coated 35 mm glass‐bottom dishes (WillCo, The Netherlands) for 48 h and followed by 10 min fixation with 4% PFA. Cells were permeabilized with 0.1% Triton X‐100 in PBS for 20 min followed by 30 min incubation in blocking buffer. For vimentin filament visualization, a monoclonal anti‐vimentin antibody (Clone LN‐6, Sigma‐Aldrich, USA) was used. Fluorescent images were obtained using a laser scanning confocal microscope Leica TCS SP8 (Leica, Germany), and morphological parameters (i.e., cell height and area, nucleus height and area) were quantified using ImageJ software (NIH, USA).

*Western Blot*: Western blot samples were prepared by lysing the confluent SCs and ECs in RIPA buffer (50 × 10^−3^ m Tris HCl pH 8, 150 × 10^−3^ m NaCl, 1% NP‐40, 0.5% sodium deoxycholate, 0.1% sodium dodecyl sulfate (SDS)). The protein concentration was measured with a Pierce BCA protein Assay kit (Thermo Fisher Scientific) and adjusted to uniform protein content, supplemented with an equal volume of 2× Laemmli sample buffer and separated on a 10% SDS‐polyacrylamide gel. Western blotting was performed using primary anti‐Lamin A antibody (Santa Cruz Biotechnology, sc‐7292) diluted 1:1000 and anti‐nucleoporin MAb414 (BioLegend, 902901) diluted 1:1000. Secondary peroxidase‐conjugated antibody (Dako, P0447) was diluted 1:3000 and detection performed using SuperSignal West‐Femto ECL detection reagent (Thermo Fisher Scientific, 34095).

*Statistical Analysis*: All experiments were repeated at least four times. Data are presented as mean values ± standard error of the mean (SEM). Results are considered as statistically significant at the probability level *P* < 0.05. The number of cells, the applied statistical tests, and the resulting *P* values are all mentioned separately in their corresponding parts. Statistical tests and graph production were performed using software Origin Pro 9.

## Conflict of Interest

The authors declare no conflict of interest.
